# Bronchoalveolar Lavage Cytology Characteristics and Seasonal Changes in a Herd of Pastured Teaching Horses

**DOI:** 10.3389/fvets.2019.00074

**Published:** 2019-03-14

**Authors:** Kaori Uchiumi Davis, Mary Katherine Sheats

**Affiliations:** ^1^Department of Clinical Sciences, College of Veterinary Medicine, North Carolina State University, Raleigh, NC, United States; ^2^Comparative Medicine Institute, North Carolina State University, Raleigh, NC, United States

**Keywords:** equine asthma syndrome, heaves, inflammatory airway disease, recurrent airway obstruction, asthma phenotype, round bale hay

## Abstract

Equine asthma syndrome (EAS) is a common problem that affects horses of any age. Severe EAS is reported to affect 10–20% of adult horses in the northern hemisphere, while mild/moderate EAS is reported to affect 60–100% of adult horses, depending on the population and geographic region. For both severe and mild/moderate EAS, the presence of lower airway inflammation is attributed to airborne “triggers” such as dust, mold, and bacterial components that horses encounter in hay and stable-environments; and treatment recommendations for horses with EAS often include full-time pasture turnout. The caveat to this recommendation is horses with summer-pasture associated EAS (SP-EAS), who experience allergic lower airway inflammation when exposed to summer pasture. The prevalence of EAS in horses on pasture that do not have SP-EAS has not been reported. The purpose of this study was to use bronchoalveolar lavage (BAL) cytology to determine the prevalence of EAS in a herd of pastured, adult research horses with no history of respiratory disease. The horses were members of a teaching animal herd housed on pasture in the southeastern United States and fed round-bale Bermuda-grass hay. BAL fluid (BALF) cytology was analyzed in both summer (May–August 2017) and winter (November 2017–February 2018). Similar to previous reports, the prevalence of severe EAS in our study population was 10% in summer and 4.3% in winter. The prevalence of mild/moderate EAS was 60% in summer and 87% in winter. The high prevalence of mild/moderate EAS in this population was unexpected, given the 24-h, year-round pasture environment and the lack of history of respiratory disease. Additionally, 61.1% of horses with both summer and winter data had a different BALF cytology profile between the two seasons. To the authors' knowledge, this is the first study to use BAL cytology to diagnose, and monitor changes in, EAS phenotype in pastured adult horses. These results help to inform discussions regarding prevalence of EAS in pastured, adult horses in the southeastern region of North America.

## Introduction

Equine asthma syndrome (EAS) is a non-infectious, inflammatory respiratory disease that includes a mild/moderate form, historically referred to as inflammatory airway disease (IAD), and a severe form, historically referred to as recurrent airway obstruction (RAO), heaves or summer pasture associated recurrent airway obstruction (SPRAO) (1). Both mild/moderate and severe EAS are characterized by lower airway inflammation and excessive mucus in the airways. While severe EAS is often diagnosed by history and clinical signs alone, bronchoalveolar lavage fluid (BALF) cytology, or other advanced diagnostic such as pulmonary function testing, is needed to diagnose mild/moderate EAS. BALF cytology is also used to monitor lower airway inflammation in response to environmental management and medication in horses with severe EAS.

BALF cytology from horses with severe asthma is characterized by moderate to severe neutrophilia (≥20–25%) as well as decreased % lymphocytes and % macrophages. BALF cytology from horses with mild/moderate EAS usually reveals mild to moderate increase in % neutrophils, % eosinophils, and/or % mast cells (1); however, different phenotypes have been recognized and associated with varying clinical signs and proinflammatory cytokine expression patterns. Specifically, an increase in % mast cells has been associated with airway hyperreactivity and altered pulmonary function (2, 3) as well as increase in IL-4 and IL-5 (4) while an increase in % neutrophils has been associated with cough (2) and increase in IL-17 and IL-23 (4, 5).

The pathophysiology of EAS is complex and significant research efforts have focused on trying to understand the contribution of multiple factors including genetics (6, 7), environment (8–11) and viral infection (12, 13). Stabling of horses has been reported as a risk factor (14), as non-infectious aerosolized particles are thought to play a key role in the development of EAS. However, the prevalence of mild/moderate and severe lower airway inflammation in horses that are not stabled has not been reported. In this study, we used BALF cytology to determine the prevalence of EAS in a university owned equine teaching herd in summer and winter. The horses were housed on pasture year-round at a veterinary teaching hospital and had no history of respiratory disease. Our hypothesis was that airway inflammation would be uncommon in pastured horses with no history of respiratory disease, and that BALF cytology would be consistent in individual horses regardless of season.

## Materials and Methods

The experimental design of this investigation was an observational, cross-sectional study. All samples were collected between May and August 2017 (summer samples) and between November and February 2018 (winter samples).

### Horses

The North Carolina State University Institutional Animal Care and Use Committee (IACUC #16-074-O) approved all procedures performed for the purposes of this study. Twenty horses were included in the summer and 25 horses were included in the winter. Horses included in this study were university owned teaching animals with no history of respiratory disease. Horses ranged in age from 6 to 24 years and were of mixed breed and gender. The horses were kept on pasture in North Carolina (southeastern United States) and received no medications for the duration of this study. Each horse received a physical exam, clinical score (Table 1) and BAL. The clinical score rubric was similar to those previously described, with slight modifications (15, 16).

**Table 1:** Clinical score definition.

	**0**	**1**	**2**	**3**
Cough score	None	Coughs at specific times of day (feeding/exercising/making beds)	Frequent cough with periods of no coughing	Very frequent cough
Nostril flare score	None	Flares during inspiration (returns to normal at end of inspiration)	Flares in inspiration and exhalation (slight movement can still be seen)	Flares in inspiration and expiration (no movement can be seen)
Abdominal lift score	None	Slight flattening of ventral flank	Obvious abdominal flattening and “heave line” extending no more than halfway between cubital joint and tuber coxae	Obvious abdominal lift and “heave line” extending beyond halfway between cubital joint and tuber coxae
Pulmonary auscultation score	Normal	Audible at inspiration and exhalation (harsh sound)	With crackles or wheezes	With crackles and wheezes
Nasal discharge score	None	Serous (scarce or abundant)	Mucous (scarce)	Mucous (abundant)

### BALF Collection and Sample Processing

Horses were sedated with detomidine (0.005–0.01 mg/kg IV) and butorphanol (0.02–0.04 mg/kg IV). A cuffed catheter was passed nasotracheally until wedged in a secondary or tertiary bronchus where 300 total mL of warmed sterile saline solution was infused and re-aspirated, 150 mL at a time. The aspirated fluid was pooled and submitted for cytological analysis by blinded clinical pathologists. Direct, concentrated and cytospin preparation slides were examined, and a 300 cell differential count was performed as part of routine cytological analysis. BALF cytology was used to assign horses to one of three groups: normal, mild/moderate inflammation or severe inflammation. Criteria for BALF cytology classification was similar to previously published guidelines (1) (Table 2), with slight modification to the cutoff used for normal and severe % neutrophils. Subtypes of mild to moderate inflammation were further categorized as mild to moderate neutrophilic inflammation (7–19% neutrophils but ≤2% mast cells), mild to moderate mastocytic inflammation (≥3% mast cells but ≤6% neutrophils) and mild to moderate mixed inflammation (7–19% neutrophils and ≥3% mast cells) (2, 17, 18).

**Table 2:** Cytologic criteria and clinical sign for disease classification.

**Normal**	**Mild/moderate neutrophilic**	**Mild/moderate mastocytic**	**Mild/moderate mixed**	**Severe**
≤6% Neutrophils ≤2% Mast cells ≤1% Eosinophils	7–19% Neutrophils ≤2% Mast cells	≥3% Mast cells ≤6% Neutrophils	7–19% Neutrophils ≥3% Mast cells	≥20% Neutrophils or ↑ Respiratory rate/effort at rest

*Fractional numbers were rounded up to the closest whole number*.

### Statistical Analysis

Clinical characteristics and BALF cytology results were analyzed by One-way ANOVA with Tukey's *post-hoc* test, except for sex, which was compared by Chi-square test. BALF total and differential cell counts were compared between summer and winter by two-tail student *t*-test, and prevalence of each disease category was compared by Fisher's exact test (due to small sample size), except for mild/moderate neutrophilic inflammation, which was compared by Chi-square test. *P* < 0.05 were considered significant. All analyses were performed using GraphPad Prism (Version 7.0 and 8.0, GraphPad Software, La Jolla, CA).

## Results

### Summer Samples

**Figure 1:**
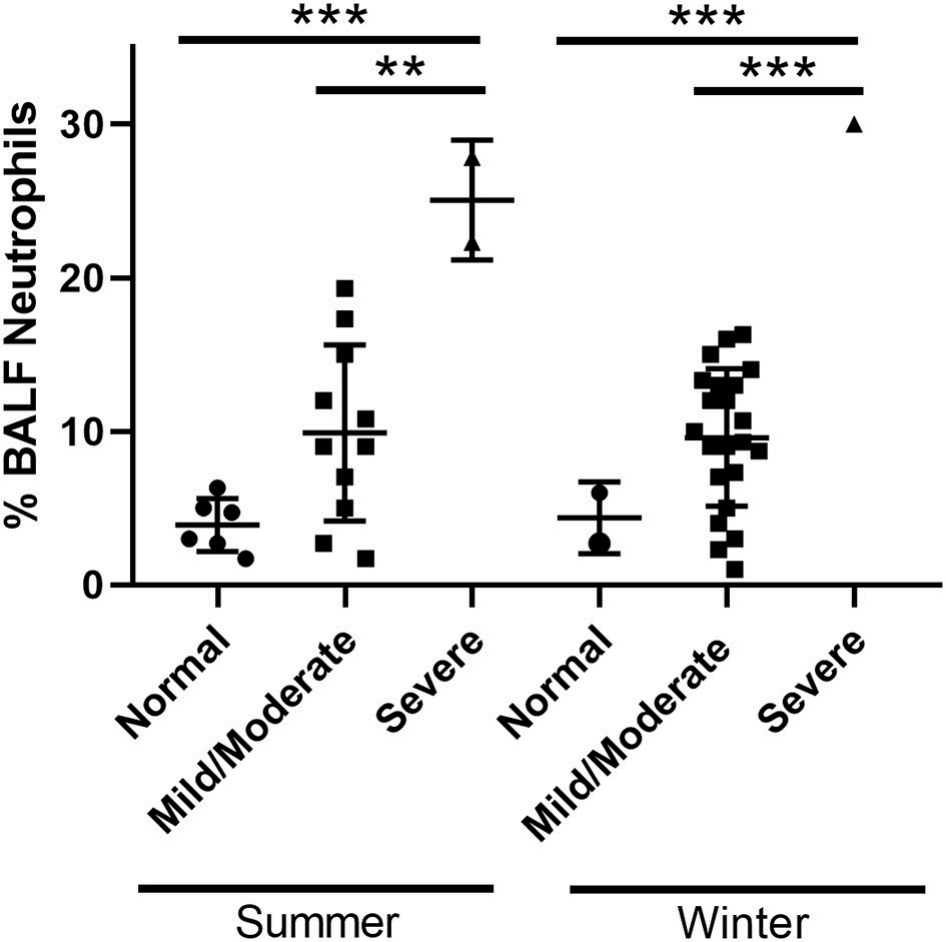
Interindividual variability. BALF neutrophil percentages of horses in each disease category. ^**^p < 0.005 and ^***^p < 0.0005.

Twenty horses were sampled during the summer period. The study population and clinical data are summarized in Table 3A. Of the 20 horses sampled, 6 (30%) had normal BALF cytology, 12 (60%) had mild/moderate inflammation and 2 (10%) had severe inflammation. There was no significant difference in the mean age among groups. Although more mares were sampled compared to geldings, the sex distribution was not significantly different among groups. There was no significant difference in mean clinical scores between groups. The mean ± SD BALF % neutrophils was significantly different in the severe group vs. normal and mild/moderate groups (*p* < 0.0005). Interindividual variability of % neutrophils in each disease category is illustrated in Figure 1. The mean ± SD % mast cells of the mild/moderate group was significantly different compared to mean % mast cells for the normal and severe groups (*p* < 0.05).

**Table 3:** Clinical characteristics and BALF cytology results.

	**Normal**	**Mild/moderate asthma**	**Severe asthma**	***p*–value**
**A. CLINICAL CHARACTERISTICS AND BALF CYTOLOGY RESULTS, SUMMER SAMPLES**				
Number of horses	6	12	2	
Mean (range) age, years	14 (9–20)	14.3 (6–24)	20 (19–21)	0.3442
Sex, mare/gelding	6/0	9/2	2/0	0.4436
Mean (range) resting respiratory rate	18.7 (12–28)	19.6 (12–32)	17 (16–18)	0.8114
Mean (range) clinical score	1.2 (0–3)	0.7 (0–2)	0.5 (0–1)	0.51
Mean (range) total cell count, /uL	351 (240–588)	296.8 (118–445)	345 (280–410)	0.6198
Neutrophils, mean (range) %	3.9 (1.7–6.3)	9.9 (1.7–19.3)	25.1 (22.3–27.8)^◇^^†^	**0.0002**
Mast cells, mean (range) %	1.4 (0.3–2.3)	3.9 (0.7–8)^‡^	2.3 (2.3–2.3)	**0.0486**
Eosinophils, mean (range) %	0.4 (0–1)	0.7 (0–3)	0.3 (0.3–0.3)	0.7238
Macrophages, mean (range) %	46.4 (37–57.3)	47.9 (28.7–66)	32.5 (31.3–33.6)	0.1924
Lymphocytes, mean (range) %	47.7 (38.7–56.3)	37.6 (16–48.3)	39.9 (36–43.7)	0.0834
**B. CLINICAL CHARACTERISTICS AND BALF CYTOLOGY RESULTS, WINTER SAMPLES**				
Number of horses	2	22	1	
Mean (range) age, years	15 (9–21)	14 (6–27)	18 (N/A)	0.7754
Sex, mare/gelding	2/0	20/2	1/0	0.8622
Mean (range) resting respiratory rate	20 (16–24)	15.1 (8–28)	12 (N/A)	0.2724
Mean (range) clinical score	2 (0–4)	1 (0–6)	1 (N/A)	0.6372
Mean (range) total cell count, /uL	389 (320–458)	359 (85–928)	235 (N/A)	0.8386
Neutrophils, mean (range) %	4.4 (2.7–6)	9.6 (1–16.3)	30 (N/A)^◇^^†^	**0.0003**
Mast cells, mean (range) %	1.35 (1–1.7)	3.0 (0–7.7)	2.7 (N/A)	0.6048
Eosinophils, mean (range) %	0 (0–0)	0.2 (0–1.7)	0 (N/A)	0.7998
Macrophages, mean (range) %	37.2 (37–37.3)	45.2 (33–72)	41 (N/A)	0.4434
Lymphocytes, mean (range) %	57.2 (56–58.3)	42 (19–54)	26.3 (N/A)	**0.0209**

*All p–values were calculated with One-way ANOVA test except for that of sex, which was calculated with Chi-square test*.

◇ *p < 0.0005 Tukey's vs. normal*.

† *p < 0.005 Tukey's vs. mild/moderate*.

‡ *p < 0.05 Tukey's vs. normal*.

*The bold values are p values where there was a statistical significance*.

### Winter Samples

Twenty-three horses were sampled during the winter period. The study population and clinical data are summarized in Table 3B. Of the 23 horses sampled, 2 (8.7%) had normal BALF cytology, 20 (87%) had mild/moderate inflammation and 1 (4.3%) had severe inflammation. There was no significant difference in the mean age among groups. Similar to the summer samples, more mares were sampled compared to geldings, but the sex distribution was not significantly different among groups. No significant difference was found in mean clinical scores between groups. The mean ± SD % neutrophils was significantly different in the severe group vs. normal and mild/moderate groups (*p* < 0.0005). Interindividual variability of % neutrophils in each disease category are illustrated in Figure 1. The mean ± SD % lymphocytes of the severe group was significantly different compared to that for the normal group (*p* < 0.05). This decrease in % lymphocytes has been reported previously in horses with severe EAS (1), and is likely due to the increased % neutrophils in the severe group.

### Seasonal Comparison

Eighteen horses had data from both summer and winter. Overall, there was a significant difference in the prevalence of mild/moderate neutrophilic inflammation in summer vs. winter samples (*p* = 0.0295). Additionally, based on BALF cytology, 11 (61.1%) horses had a different disease classification or subtype classification between the two seasons, while the BALF cytology profiles remained consistent in 7 (38.9%) horses. The disease classifications are summarized in Table 4.

**Table 4:** Disease classifications of the 18 horses with both summer and winter data.

**Horse ID**	**Classification**	
	**Summer**	**Winter**
1	Mild/Moderate neutrophilic	Mild/Moderate neutrophilic
2	Mild/Moderate neutrophilic	Mild/Moderate neutrophilic
3	Mild/Moderate neutrophilic	Mild/Moderate neutrophilic
4	Mild/Moderate mixed	Mild/Moderate mixed
5	Mild/Moderate mixed	Mild/Moderate mixed
6	Mild/Moderate mixed	Mild/Moderate mixed
7	Normal	Normal
8	Normal	Mild/Moderate neutrophilic
9	Normal	Mild/Moderate neutrophilic
10	Normal	Mild/Moderate neutrophilic
11	Normal	Mild/Moderate neutrophilic
12	Normal	Mild/Moderate mastocytic
13	Mild/Moderate mastocytic	Mild/Moderate neutrophilic
14	Mild/Moderate mastocytic	Severe
15	Mild/Moderate mixed	Mild/Moderate neutrophilic
16	Mild/Moderate mixed	Mild/Moderate neutrophilic
17	Severe	Mild/Moderate neutrophilic
18	Severe	Normal

## Discussion

Severe EAS is reported to affect 10–20% of adult horses in the northern hemisphere and other temperate climates (19, 20). In our study population, 2 of 20 (10%) horses in the summer, and 1 of 23 (4.3%) in the winter, had BALF cytology consistent with severe EAS. One horse had severe EAS in the summer but normal BALF cytology in the winter, suggesting a diagnosis of summer-pasture associated EAS (SP-EAS, also referred to as summer pasture associated recurrent airway obstruction or SPRAO), which had not been previously diagnosed. While the prevalence of severe EAS in our teaching horse population was similar to previous reports, we were surprised by the high prevalence of mild/moderate EAS, given the lack of history of respiratory disease in these horses, and their 24-h pasture environment. Potential explanations for the high prevalence of mild/moderate lower airway inflammation in this herd include: continuous access to round bale hay and exposure to air pollution due to pasture location in an urban setting and close proximity of pasture to a heavily traveled 4-lane road. Hay feeding causes increased particulate in the breathing zone, which can lead to airway inflammation (21, 22). Additionally, the method of hay processing also affects the airway health of horses. Horses fed round bale hay are known to have an elevated risk of airway inflammation compared to those fed square bale hay (23, 24). This finding is due to higher numbers of thermophilic actinomycetes found in round bale hay (25) and increased dust exposure for horses that keep their faces immersed in the round bale while eating. Air pollution is another cause of increased particulate concentration. In people, there is a strong association between air pollutant levels and increased frequency and severity of asthma attacks (26–28). Similar to humans, traffic pollution has also been associated with airway inflammation in horses (10).

Compared with severe EAS, fewer studies have investigated the prevalence and/or environmental factors associated with mild/moderate EAS. In one study comparing airway inflammation and mucus in two age groups of sport horses housed in a conventional stable environment, 100% of the horses had increased BALF % neutrophils and/or % mast cells, despite the lack of history or clinical signs of respiratory disease (29). In an observational study in a population of racing thoroughbreds, evidence of mild EAS was found in 80% (78/98) of BALF samples from 52 out of 64 horses (15). In these studies, as well as others, investigators conclude that stable dust likely contributes to the high prevalence of mild/moderate EAS in performance horses. Indeed, mild/moderate EAS is often only investigated in sport horses with performance “issues”. Therefore, the prevalence of mild/moderate EAS in non-exercised horse, and the clinical relevance of mild/moderate EAS in horses with no clinical signs or history of poor performance, is largely unknown. Results from our study suggest that non-infectious lower airway inflammation may be a common finding, even in horses not exposed to stabling environments.

**Figure 2:**
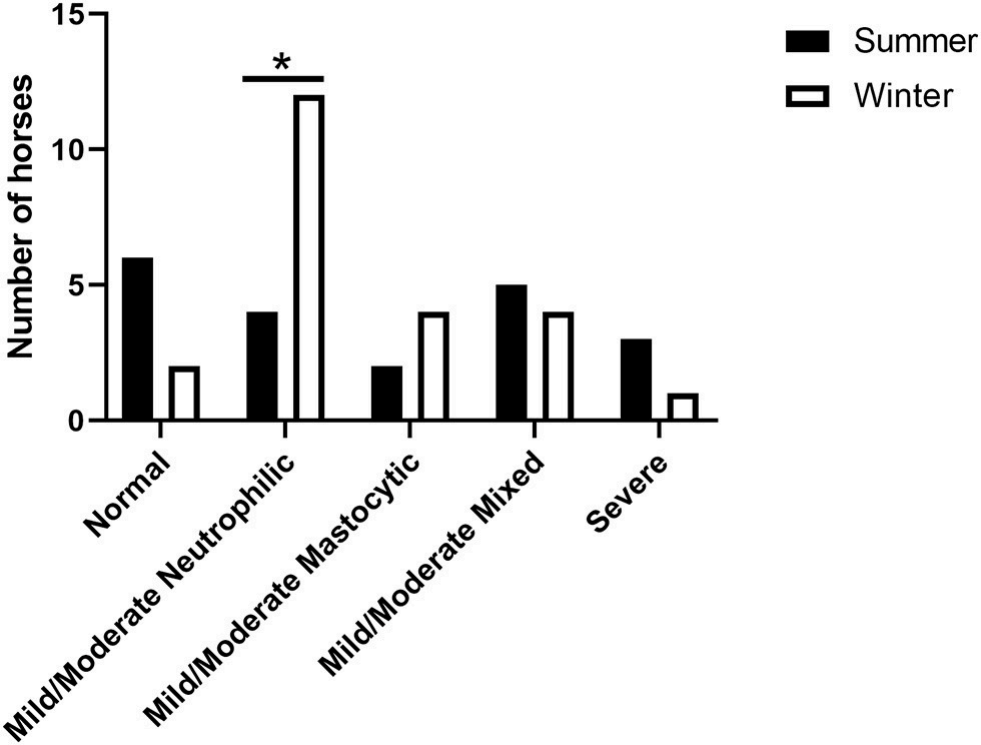
Seasonal distribution of categorized inflammatory subtype. The prevalence of mild/moderate neutrophilic inflammation was significantly higher (^*^p < 0.05) in winter compared to summer.

In our study population, the neutrophilic subtype of mild/moderate EAS was more prevalent in winter (Figure 2). Increased BALF % neutrophils in winter has been reported in other population of horses as well. In a previous report, Riihimaki et al. evaluated differences in air quality and markers of airway inflammation by season and found a trend toward elevation of BALF % neutrophils during winter. Riihimaki et al. also report increased expression of IL-6 mRNA in BAL cells and inhalable dust and 1,3-β-glucan in the breathing zone during winter (11). Similarly, Hansen et al. report significantly higher BALF % neutrophils and mucus score in November compared to May (30). In these studies investigators suggest that client-owned horses are stabled more in winter, leading to an increase in exposure to airborne irritants and a concomitant airway neutrophilia. In our population, however, increased stabling was not a potential cause of increased lower airway neutrophils, as the horses in our study group were kept on pasture year-round.

One possible explanation for the seasonal difference in BALF cytology profile is change in temperature and humidity. Cold and dry air is known to exacerbate asthma. Asthma-like respiratory symptoms and chronic airway inflammation are reported by athletes who perform repeated exercise while breathing cold air (termed “ski asthma”) (31, 32). Airway dehydration is reported to trigger bronchoconstriction, and altered airway hydration is associated with airway neutrophilia and increased levels of IL-8 (33). In dogs, persistent airway neutrophilic and eosinophilic inflammation, obstruction and hyperreactivity have been demonstrated following challenge with cold and dry air (34). Similarly, in two studies in healthy adult exercising horses, Davis et al. report that horses that breathe cold air during exercise experience significant upregulation of TH2 cytokines IL-4, IL-5, and IL-10 (35) and proinflammatory cytokines IL-1, IL-6 and IL-8 in BALF cells, as well as a significant increase in the BALF % neutrophils (36). Another possible explanation for the seasonal difference we observed in airway neutrophilia is change in hay consumption. Horses spend increased amount of time eating from round bale hay during cold months due to dormant pasture and increased caloric demand, both of which result in increased exposure to dust and molds. Taken together, seasonal changes in ambient air temperature and humidity, as well as increased consumption of round bale hay, may be contributing to the increased neutrophilic inflammation we observed in our population in the winter.

In addition to investigating the prevalence of EAS in our teaching herd, we also sought to determine whether BAL cytology from individual horses was consistent over time. Eleven out of 18 horses (61.1%) in our study group with both summer and winter BALF cytology data had differing cytology profiles in summer vs. winter. This finding was the opposite of what we hypothesized and somewhat surprising, given the consistent environment of our study. Previous reports on the consistency of BALF cell differential counts in individual horses are conflicting. BAL cytology results reported by Thomas et al. show diagnostically relevant differences in BALF % neutrophils in ponies in clinical remission from heaves, as well as healthy ponies, when samples were collected 2–15 weeks apart (37); while findings from Jean et al. show consistent BAL cytology from horses sampled monthly for a period of 3 months (38). Differences in housing could explain the apparent discrepancies in these reports, as ponies in the Thomas et al. study were housed on pasture and horses in the Jean et al. study were stabled. Another important factor to consider when assessing the prevalence of lower airway inflammation in horses is variation during BAL procedure and sample processing that contribute to cytologic findings. Multiple variables including volume of fluid instilled (38, 39), number of aliquots collected (38, 40), sampling site (38, 41), slide preparation (42), and cell counting methods (43) are reported to affect differential cell counts, making characterization of mild to moderate lower airway inflammation challenging. The resulting variability in cytologic findings makes it difficult to establish definitive cutoff values for the classification of EAS. Nevertheless, results reported here, combined with findings reported by Thomas et al. could indicate that the BALF cytology profile of pastured horses may be more dynamic than stabled horses, and that one-time BALF cytology may be insufficient to characterize the complete picture of the airway immune response in horses on pasture.

Our study had several limitations. First, because our herd was small, further investigations will be needed to determine whether these results extrapolate to other population of horses. Second, disease classification in this study was solely based on BALF cytology, and further analyses such as pulmonary function testing and/or endoscopy were not available. Therefore, it is unclear how BALF cytology in our study population correlates with lung function or airway hyperreactivity in these horses. Finally, we do not know if any of the horses in our population have inducible, reversible bronchoconstriction, as antigen challenge was not performed. Further research will be needed to determine whether individual horse variability in BAL cytology correlates with disease status as determined by pulmonary function testing or antigen challenge. Despite these limitations, our study findings provide important data on the prevalence of EAS in a different type of horse population than those previously examined. We hope this data will provide more context for equine practitioners who perform BALF cytology and are required to interpret their findings without the aid of advanced diagnostics.

While the prevalence of severe EAS in our study population is similar to previous reports, we were surprised by the 60–87% prevalence of mild/moderate EAS in pastured horses with no history of respiratory disease. We also did not expect an increase in mild/moderate neutrophilic inflammation in the winter. To the authors' knowledge, this is the first study to describe the prevalence of EAS, as well as seasonal changes in BALF cytology profiles, in pastured horses. We expect these results to help inform equine clinicians and researchers regarding expectations and interpretation of BALF cytology for horses in different environments and during different seasons. In future studies, we will utilize more advanced diagnostics (i.e., conventional pulmonary function testing) to determine whether classification by BALF cytology correlates with inducible bronchoconstriction in our herd. Additionally, we will determine whether lower airway inflammation in these horses is responsive to environmental management (i.e., removal of round bale-hay).

## Data Availability

All datasets generated for this study are included in the manuscript and/or the supplementary files.

## Author Contributions

KD was responsible for study design, experimental execution, and preparing the manuscript. MS was responsible for overseeing all aspects of the study, including study design, and critically reviewing the manuscript.

### Conflict of Interest Statement

The authors declare that the research was conducted in the absence of any commercial or financial relationships that could be construed as a potential conflict of interest.

(1) $$\begin{array}{l} E(A,s)=\frac{1}{2}\|x-As{\|}_{2}^{2}+\lambda \sum_{i}Q(s_{i}), \end{array}$$

(2) $$\begin{array}{l} \;\dot{s}=A^{T}r-\lambda Q\prime (s)\\ \Delta A\propto \langle rs^{T}\rangle , \end{array}$$

(3) $$\begin{array}{l} \tau_{\text{L}}{\dot{v}}_{\text{ON}}^{\text{L}}\;=-v_{\text{ON}}^{\text{L}}+x_{\text{ON}}+A_{\text{ON}}^{\text{d},\text{+}}s^{\text{C}}+A_{\text{ON}}^{\text{d},\text{−}}s^{\text{C}}+s_{\text{b}}\\ \;s_{\text{ON}}^{\text{L}}=\max(v_{\text{ON}}^{\text{L}},0) \end{array}$$

(4) $$\begin{array}{l} \tau_{\text{L}}{\dot{v}}_{\text{OFF}}^{\text{L}}=-v_{\text{OFF}}^{\text{L}}+x_{\text{OFF}}+A_{\text{OFF}}^{\text{d},\text{+}}s^{\text{C}}+A_{\text{OFF}}^{\text{d},-}s^{\text{C}}+s_{\text{b}},\\ \;s_{\text{OFF}}^{\text{L}}=\max(v_{\text{OFF}}^{\text{L}},0), \end{array}$$

(5) $$\begin{array}{l} \tau_{\text{L}}{\dot{v}}^{\text{L}}=-v^{\text{L}}+x+(A^{\text{d},\text{+}}+A^{\text{d},-})s^{\text{C}}+s_{\text{b}}\\ \;s^{\text{L}}=\max(v^{\text{L}},0). \end{array}$$

(6) $$\begin{array}{l} \tau_{\text{C}}{\dot{v}}^{\text{C}}=-(v^{\text{C}}-v_{\text{leak}}^{\text{C}})+A_{\text{ON}}^{u,+T}s_{\text{ON}}^{\text{L}}+A_{\text{ON}}^{u,-T}s_{\text{ON}}^{\text{L}}\\ \;+A_{\text{OFF}}^{u,+T}s_{\text{OFF}}^{\text{L}}+A_{\text{OFF}}^{u,-T}s_{\text{OFF}}^{\text{L}}+s^{\text{C}}, \end{array}$$

(7) $$\begin{array}{l} \tau_{\text{C}}{\dot{v}}^{\text{C}}=-v^{\text{C}}+v_{\text{leak}}^{\text{C}}+{\left(A^{\text{u},\text{+}}+A^{\text{u},-}\right)}^{T}s^{\text{L}}+s^{\text{C}}\\ \;s^{\text{C}}=\max(v^{\text{C}}-\lambda ,0), \end{array},$$

(8) $$\begin{array}{l} \Delta A^{\text{u},\text{+}}=\eta \langle (s^{\text{L}}-s_{\text{b}})s^{{\text{C}}^{T}}\rangle \\ \Delta A^{\text{u},-}=\eta \langle (s^{\text{L}}-s_{\text{b}})s^{{\text{C}}^{T}}\rangle \\ \Delta A^{\text{d},\text{+}}=-\eta \langle (s^{\text{L}}-s_{\text{b}})s^{{\text{C}}^{T}}\rangle \\ \Delta A^{\text{d},-}=-\eta \langle (s^{\text{L}}-s_{\text{b}})s^{{\text{C}}^{T}}\rangle , \end{array}$$

(9) $$\begin{array}{l} R(f)=fe^{-{\left(f/f_{c}\right)}^{4}}, \end{array}$$

(10) $$\begin{array}{l} F=\frac{s_{1}n_{1}+\cdots +s_{K}n_{K}}{s_{1}+\cdots +s_{K}}. \end{array}$$

(11) $$\begin{array}{l} L(f)=e^{-{\left(f/f_{s}\right)}^{4}}. \end{array}$$

(12) $$\begin{array}{l} G(x,y;x_{0},y_{0},\sigma_{x},\sigma_{y},f_{s},\beta ,\theta ,\phi )\\ \;=\beta \cos(2\pi f_{s}x^{\prime }+\phi )e^{-{\left(\frac{x^{\prime }}{\sqrt{2}\sigma_{x}}\right)}^{2}-{\left(\frac{y^{\prime }}{\sqrt{2}\sigma_{y}}\right)}^{2}} \end{array}$$

(13) $$\begin{array}{l} x^{\prime }=(x-x_{0})\cos\theta +(y-y_{0})\sin\theta \\ y^{\prime }=-(x-x_{0})\sin\theta +(y-y_{0})\cos\theta , \end{array}$$

(14) $$\begin{array}{l} h(x,y;x_{0},y_{0},a,b,\theta ,\gamma )=\frac{\gamma }{2\pi ab}e^{-\frac{{x^{\prime }}^{2}}{2a^{2}}-\frac{{y^{\prime }}^{2}}{2b^{2}}} \end{array}$$

(15) $$\begin{array}{l} I_{\text{o}}=\frac{W_{\text{ON}}+W_{\text{OFF}}-d}{W_{\text{ON}}+W_{\text{OFF}}+d},\;(-1<I_{\text{o}}\leq 1) \end{array}$$

(16) $$\begin{array}{l} P=\frac{P}{\max(|P|,|N|)}\text{ and }N=\frac{N}{\max(|P|,|N|)}. \end{array}$$

(17) $$\begin{array}{l} I_{\text{p}}=|P+N|,\;(0\leq I_{\text{p}}\leq 2). \end{array}$$

(18) $$\begin{array}{l} S_{\text{f}}=(A_{\text{ON}}^{\text{u},\text{+}}+A_{\text{ON}}^{\text{u},-})-(A_{\text{OFF}}^{\text{u},\text{+}}+A_{\text{OFF}}^{\text{u},-}) \end{array}$$

(19) $$\begin{array}{l} \Delta f:=h(n_{x})=\log_{2}\left(\frac{1+\frac{\sqrt{2\ln2}}{2\pi n_{x}}}{1-\frac{\sqrt{2\ln2}}{2\pi n_{x}}}\right)\text{ in octaves}\\ \Delta \theta :=g(n_{y})=2\arctan\left(\frac{\sqrt{2\ln2}}{2\pi n_{y}}\right)\text{ in degrees}. \end{array}$$

(20) $$\begin{array}{l} \tau_{\text{C}}{\dot{v}}^{\text{C}}=-v^{\text{C}}+A^{T}(s^{\text{L}}-s_{\text{b}})+s^{\text{C}}. \end{array}$$

(21) $$\begin{array}{l} \tau_{\text{L}}{\dot{v}}^{\text{L}}=-v^{\text{L}}+x-As^{\text{C}}+s_{\text{b}}. \end{array}$$
